# Correction to: Morphometric feature description of the proximal ulna based on quantitative measurement: a key consideration for implant design

**DOI:** 10.1007/s00276-022-03071-x

**Published:** 2022-12-29

**Authors:** Daofeng Wang, Jiantao Li, Gaoxiang Xu, Hao Zhang, Cheng Xu, Wupeng Zhang, Hua Li, Xuewen Gan, Ying Xiong, Licheng Zhang, Li Li, Peifu Tang

**Affiliations:** 1 Medical School of Chinese PLA, Beijing, China; 2 Department of Orthopedics, The Fourth Medical Center of Chinese PLA General Hospital, No. 51 Fucheng Road, Beijing, 100048 China; 3 National Clinical Research Center for Orthopedics, Sports Medicine and Rehabilitation, Beijing, China; 4 Department of Orthopedics, School of Medicine, Nankai University, Tianjin, China; 5 Department of Orthopedics, Kunming Medical University, Yanan Hospital, Kunming, China

**Correction to: Surgical and Radiologic Anatomy** 10.1007/s00276-022-03058-8

The article “Morphometric feature description of the proximal ulna based on quantitative measurement: a key consideration for implant design”, written by Daofeng Wang, Jiantao Li, Gaoxiang Xu, Hao Zhang, Cheng Xu, Wupeng Zhang, Hua Li, Xuewen Gan, Ying Xiong, Licheng Zhang, Li Li, Peifu Tang was originally published online on the publisher’s internet portal on 12 December 2022 with Open Access under a Creative Commons Attribution (CC BY) license 4.0. With the author’s/authors’ decision to cancel Open Access the copyright of the article changed on 21st December 2022 to © Springer-Verlag France SAS, part of Springer Nature 2022 with all rights reserved.

